# Assessment of oral health status in a Ghanaian population: rationale, methods, and population characteristics

**DOI:** 10.1186/s12903-022-02090-9

**Published:** 2022-03-12

**Authors:** Sandra Ama Hewlett, Paa-Kwesi Blankson, Justice Moses K. Aheto, Francis Anto, Tony Danso-Appiah, Josephine Sackeyfio, Kwadwo Koram, Albert G. B. Amoah

**Affiliations:** 1 Department of Restorative Dentistry, University of Ghana Dental School, College of Health Sciences, University of Ghana, Accra, Ghana; 2 Department of Oral and Maxillofacial Surgery, University of Ghana Dental School, College of Health Sciences, University of Ghana, Accra, Ghana; 3 Oral and Maxillofacial Unit, Korle-Bu Teaching Hospital, Accra, Ghana; 4 Department of Biostatistics, School of Public Health, College of Health Sciences, University of Ghana, Accra, Ghana; 5 Department of Epidemiology and Disease Control, School of Public Health, College of Health Sciences, University of Ghana, Accra, Ghana; 6 Department of Community and Preventive Dentistry, University of Ghana Dental School, College of Health Sciences, University of Ghana, Accra, Ghana; 7 Noguchi Memorial Institute for Medical Research, College of Health Sciences, University of Ghana, Accra, Ghana; 8 Department of Medicine and Therapeutics, University of Ghana Medical School, College of Health Sciences, University of Ghana, Accra, Ghana

**Keywords:** Ghana, Oral health, Survey, Dental caries, Periodontal disease, Retained roots

## Abstract

**Background:**

Oral health surveys aid in estimating the oral health of a population and provide a projection for future oral health care needs. We report the procedures and rationale of a survey carried out to assess the oral health status and risk factors for oral disease among adults in the Greater Accra Region (GAR) of Ghana. The objective was to provide prevalence estimates on dental diseases, oral health behaviour and risk factors, and to establish baseline epidemiological data on the population’s oral health for further research.

**Methods:**

This was a population-based cross-sectional study of adults aged 25 years and above. A random, stratified two-stage sampling method was used to select participants from rural and urban communities in three types of districts (Metropolitan, Municipal, Ordinary). A semi- structured questionnaire was used to collect data on socio-demographic characteristics, oral health behaviours and risk factors for oral disease. Anthropometric data and a full-mouth clinical examination was carried out including: soft tissue assessment, tooth count, prosthodontic status, dental caries assessment and periodontal assessment.

**Results:**

A total of 729 participants were included in the study with a mean age of 43.9 years (SD 14.6). Majority 425 (61.0%) were females. Though the metropolitan districts had more dental clinics and personnel, along with better health insurance coverage, they had a higher prevalence of missing teeth, retained roots, severe periodontitis and poorer oral health coverage. The findings also show some significant differences in disease prevalence, within the different localities and districts.

**Conclusions:**

Availability and access to oral health services is not the most important determinant of good oral health outcomes in this region. We recommend exploring socio-behavioral and cultural factors as well. This study provides district level data to inform policy and guide further research.

## Introduction

Oral disease poses a major public health burden for many countries and affects individuals throughout their lifetime, causing pain, disfigurement, impairment of function and a reduced quality of life [1]. It has a high prevalence globally, collectively being the commonest chronic disease worldwide [2] and yet it remains underestimated. The distribution and severity of oral disease has also shown considerable variation worldwide, and within countries. These disparities are related to living conditions, behavioural and environmental factors, oral health systems and preventative health schemes, with a disproportionate burden borne by disadvantaged populations [3]. In Africa, the profile of oral diseases is also not homogeneous and though data is scarce, available evidence suggests an increasing trend [4]. Yet oral health is seen as a very low priority and the limited resources available to the health sector are directed towards other more life-threatening conditions [5].

Ghana has had some challenges with access to dental care including low-socioeconomic status, low dentist: population ratio and a paucity of data and targeted oral health programs focused on at risk populations. Regular oral health surveys have been used to assess changes in oral health patterns and trends. This ensures that problems are identified early for appropriate and timely planning of services. The World Health Organisation (WHO) recommends that countries conduct population based oral health surveys every 5–6 years [6]. This provides decision makers with needed information about risk factors to help identify target populations for implementation of interventions. It also helps in the monitoring of the oral health of a population, while evaluating access to preventive and treatment services. This is especially essential in Africa and Ghana in particular where utilization of oral healthcare services is very low [7].

The first and only nationally representative oral health survey in Ghana was carried out in 1963 [8]. Subsequently, several studies have reported data on local communities and at-risk populations [9, 10]. The absence of current national and regional data on oral diseases is thus a significant gap in oral health service delivery in Ghana. We report a population-based oral health status assessment in the Greater Accra Region (GAR) of Ghana, the most urbanized province of the country. Its objective was to provide prevalence estimates on dental diseases and risk factors, and to establish baseline epidemiological data on the population’s oral health for further research projects.

## Methods

**Fig. 1:**
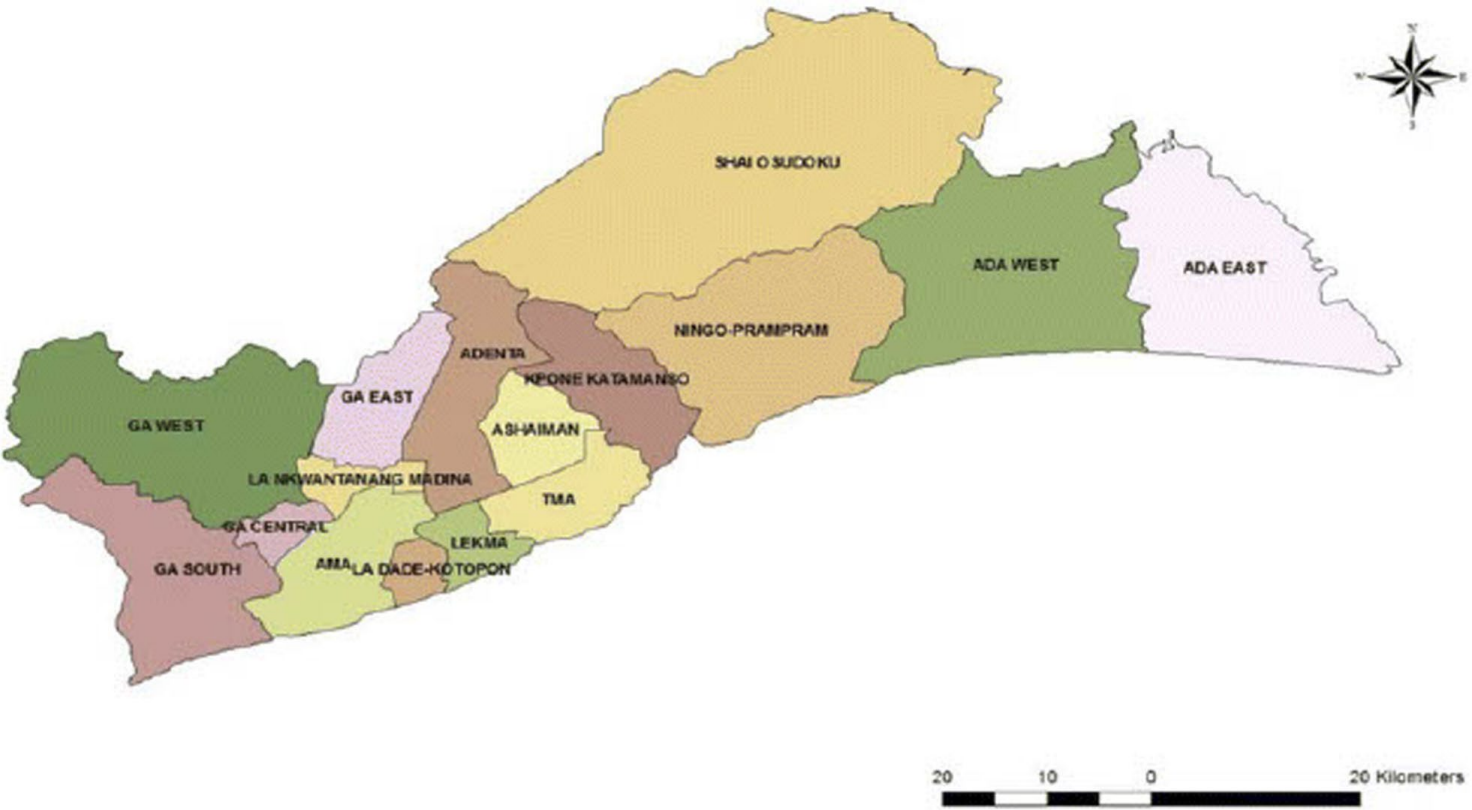
Map of the Greater Accra Region showing its 16 districts

This study was a population-based cross-sectional study carried out from June to September 2016. Ghana is a country located on the West Coast of Africa and is divided into 16 administrative regions. With a population of 4,010,054 in 2010, the GAR is the second most populated administrative region and accounts for 16.3% of Ghana's population [11]. The region is further divided into 16 districts (Fig. 1), which are sub-classified into two metropolitan, nine municipal, and five ordinary districts defined by a minimum population of 250,000, 95,000 and 75,000 respectively. Majority (90.5%) of its population live in urban localities with an annual urban growth rate of 3.1%.

The GAR was chosen for this study because it harbours the largest proportion of oral health personnel and clinics in the country. Within the region, 89.0% of the dental clinics are located in the two metropolitan areas and 5.5% each located in the municipal and ordinary districts. The region is therefore very diverse in terms of access to oral health care and is a rich source of information in comparing risk factors from respondents with good and poor access to oral healthcare. The target population was adults aged 25 years and above who reside in the GAR.

We used a stratified two-stage sample design to allow estimates of key indicators at the district level as well as urban and rural areas. The sampling frame was the 2010 population and housing census.

Sample size was estimated using a prevalence of 56.0% [10] for periodontal disease and a margin of error of 5.0% at 95.0% confidence level. An estimate of 379 was obtained but to mitigate the effect of possible sampling errors, due to the design, the standard error was increased by a factor of 1.5. Also, factoring a 25.0% non-response [12], a total of 712 was estimated. To ensure equal numbers from each EA, a total of 800 was estimated as the minimum sample to be recruited.

**Fig. 2:**
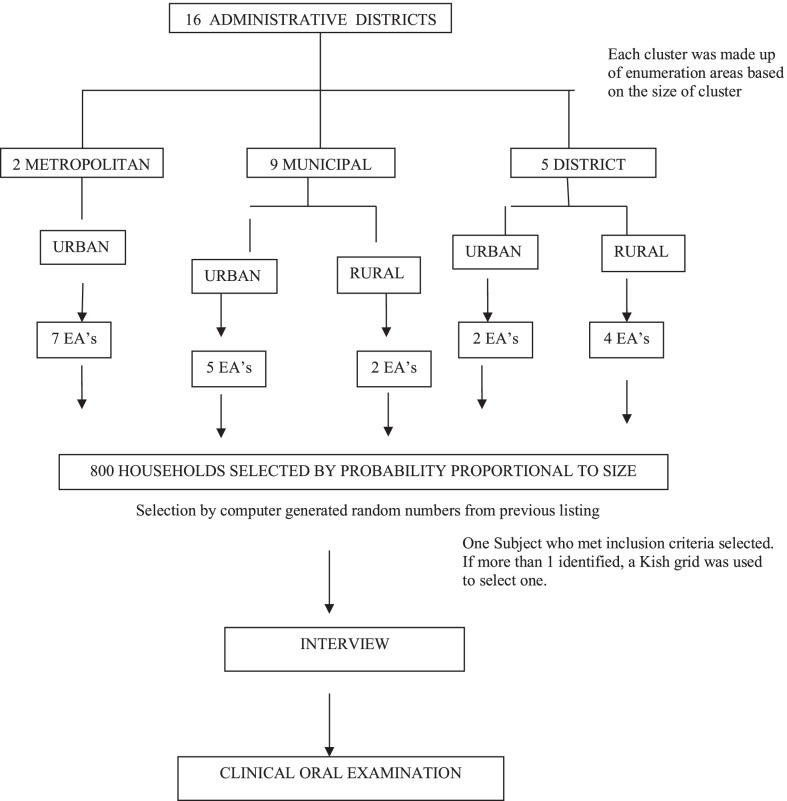
Schematic diagram of the sampling strategy used for the study

In the region, the 16 districts are each subdivided into enumeration areas (EA) with each EA being either urban or rural. We therefore stratified the region by urban and rural localities of residence, and by the three types of districts; Metropolitan, Municipal and ordinary District. A two-stage sampling methodology was used in selecting 800 households (Fig. 2). The first stage involved the random selection of 20 EA’s from the three district types, consisting of 14 urban EAs and 6 rural EAs. The selection was carried out using computer generated random numbers. At the second stage, a household listing of all households in each EA served as the sampling frame for the selection of 40 households, and one individual from each household was recruited. The households were then selected by systematic sampling proportional to size. The selected samples were, however, not self-weighting since the rural areas and the ordinary districts were over-sampled. (The metropolitan districts are all wholly urban and the ordinary districts had a smaller population). Thus a final weighting adjustment was done to provide estimates for each domain according to recommended strategy [13]. The selected participants for each EA were assembled at a prearranged venue where a mobile dental clinic with the research team carried out interviews, a physical and clinical oral examination.

### Study procedures

A semi-structured questionnaire was used to collect information on the respondents’ background characteristics, socioeconomic status, attitudes and oral health habits. Their health state was also assessed by identifying disease conditions they had been diagnosed with.

Three dentists, 3 hygienists and 6 dental surgery assistants participated in a one-week training and calibration session, at the University of Ghana Dental School (UGDS) Clinic by a National Health and Nutrition Examination Survey (NHANES) dental examiner following NHANES Guidelines [14]. To ascertain examiner agreement with the reference examiner, Kappa (κ) statistics for categorical variables, and inter-class correlation coefficients (ICC) for continuous variables were calculated. A κ score > 0.8 and ICC > 0.7 indicated good agreement. If an examiner's κ score was less than 0.8, and ICC was less than 0.7, they were re-trained and evaluated repeatedly till there was good agreement.

Following this, the whole research team including 4 interviewers underwent training on procedures for data collection.

Interviews were conducted using interviewer administered questionnaires. The questionnaires were pre-tested with 40 different participants in a different population with similar characteristics and additions and edits made to it before the study. The questions in the questionnaire was adapted from the NHANES and WHO oral health surveys and have been validated.

The questionnaire included modules on socio-demographics, risk factors for oral disease, chronic conditions and anthropometric measurements. They included:

### Sociodemographic factors

Age, sex, ethnicity, religion, marital and educational status, place of residence (urban or rural), economic and health insurance status was obtained through self-report.

### Oral health risk factors

Oral hygiene practices, dietary practices, dental attendance patterns, smoking and alcohol use. Questions to assess the presence of risk factors were based on the World Health Organization (WHO) STEPwise approach to non-communicable disease risk factor surveillance [15]. Respondents were asked about their current and past smoking habits. From these, they were categorized as being non-smokers, current smokers or former smokers. Another question, “What best describes your use of alcohol?” was used to assess alcohol use. From this, they were categorized as having never used alcohol, former users or current users. For current users, they were further categorized into regular drinkers or occasional users.

Oral healthcare coverage: was derived by utilizing two questions from the questionnaire, (1) During the last 12 months, did you have any problems with your mouth and/or teeth? (2) During the last 12 months, did you receive any medical care or treatment from a dentist or other oral health specialist for this problem with your mouth and/or teeth? Oral healthcare coverage was defined as the proportion of individuals who expressed a need (as indicated by the first question) that answered the second question positively [16].

### Anthropometric measurements

The height of the respondents was measured with a seca stadiometer and recorded in centimetres to the nearest 0.1 cm. Their weight was measured with a seca 762 weighing scale in kilograms to the nearest 0.1 kg. From these, body mass index (BMI) was computed as weight (kg)/height (metres)^2^. Obesity was defined as BMI ≥ 30 kg/m^2^, overweight as BMI ≥ 25 kg/m^2^ and < 30 kg/m^2^ and underweight as BMI < 18.5 kg/m^2^ [17].

A 203 cm non-elastic, plastic seca measuring tape with 1 mm divisions was used for the measurement of waist and hip circumferences. A high waist circumference was defined as waist circumference > 90 cm for males and > 84 cm for females. A high WHR or central obesity was defined as WHR > 0.90 for males and 0.85 for females [17].

In addition to a self-reported hypertension diagnosis, the blood pressure of all participants was measured using an *OMRON 10* series blood pressure monitor model *BP786N*. In accordance with the WHO STEPwise approach to chronic disease risk-factor surveillance protocol [15], three measurements were taken and the average of the last two readings estimated. The respondent was considered to be hypertensive if the mean of the last two measurements was ≥ 140 mmHg (systolic BP) or ≥ 90 mmHg (diastolic BP), or if the respondent was currently taking anti-hypertensive medications.

Glycosylated Haemoglobin (HbA1c) was measured using A1CNow + ®, (PTS Diagnostics, Whitestown, Indiana, USA). Diabetes was considered to be present if HbA1c was 6.5% or above, prediabetes if HbA1c was between 6.0 and 6.4% and normal if below 6.0% [18].

### Oral examination

This consisted of a general oral examination, caries assessment and a periodontal examination.

The general oral examination consisted of a soft tissue assessment involving an evaluation of the soft palate, hard palate, gingival and buccal mucosa, muco-gingival folds, tongue, sub-lingual area, sub-mandibular area, salivary glands, and tonsilar and pharyngeal area. All teeth present were counted, and their absence including the presence of prostheses and retained roots were recorded. All teeth present were also assessed for dental caries and restorations. The Decayed, Missing, and Filled Teeth (DMFT) index was used to measure the caries experience of the population. This index was the sum of the individual's decayed, missing, and filled permanent teeth with the exception of the third molars.

A full mouth periodontal examination was then conducted on six sites of all teeth excluding the third molars with a manual periodontal probe (Hu Friedy PCP UNC-12).

Periodontal status was assessed by probing pocket depth (PPD), and clinical attachment loss (CAL). Six sites per tooth were assessed. The PPD was measured as the distance in millimetres between the free gingival margin (FGM) and the base of the pocket/sulcus. To obtain CAL, gingival recession/hyperplasia (the CEJ-FGM distance) was measured as the distance between the cemento-enamel junction and the free gingival margin. CAL was then computed at the analysis stage, as the difference at each site between the measures of pocket depth and the CEJ-FGM distance.

Gingivitis was defined as the presence of gingival bleeding on probing (BOP) in at least one site [19] and periodontitis was classified according to the CDC-AAP case definition [20].

Data were entered into Microsoft Excel for cleaning, recoding, and validation for completeness and data quality. Statistical analyses were performed to summarise the data in the form of frequencies and percentages, and presented as tables. To determine the relationship between oral health conditions and the associated factors, unadjusted and adjusted binary logistic regression models adjusting for the complex survey design used were carried out. Odds ratios (OR) and their 95% confidence intervals (CI) from simple and multiple logistic regression models were reported as a measure of the strength of the association.

All the analyses were done using STATA 14 software (StataCorp. College Station, TX).

The study along with all its method and procedures have been performed in accordance with the Declaration of Helsinki. Ethical approval was obtained from the Ghana Heath Service Ethical Review Committee (GHS-ERC: 15/09/15) and the University of Ghana School of Medicine and Dentistry Ethical and Protocol Review Committee (CHS-Et/M.7-P4.7/2015–2016). Permission was obtained from the Metropolitan, Municipal or District Directors of Health Services for all the districts selected. The research was explained to all the participants after which their written informed consent was obtained from all of them before the study related procedures were carried out.

## Results

Of the 800 households sampled, 729 respondents consented to participating in the study and were interviewed and examined, resulting in a 91.0% response rate. The mean age of the respondents was 43.9 (SD 14.6) years with a range of 25 to 95 and a median of 42 years (Table 1). Majority (61.2%) of them were female with a 0.72: 1 male: female ratio.

**Table 1:** Socio-demographic characteristics of respondents by district type

Characteristic	Metropolitan (%*)	Municipal n (%*)	Ordinary District n (%*)	Pooled n (%*)
*Residence*				
Urban	251 (100)	229 (77.1)	39 (21.5)	519 (86.3)
Rural	0	68 (22.9)	142 (78.5)	210 (13.7)
*Sex*				
Male	82 (32.7)	138 (46.5)	84 (46.4)	304 (38.8)
Female	169 (67.3)	159 (53.5)	97 (53.6)	425 (61.2)
*Age group*				
25–34	58 (23.3)	112 (37.7)	60 (33.5)	230 (29.4)
35–44	59 (23.7)	73 (24.6)	43 (24.0)	175 (24.1)
45–54	59 (23.7)	53 (17.9)	30 (16.8)	142 (21.0)
55–64	45 (18.1)	36 (12.1)	24 (13.4)	105 (15.5)
65–74	20 (8.0)	20 (6.7)	10 (5.6)	50 (7.4)
75 +	8 (3.2)	3 (1.0)	12 (6.7)	23 (2.6)
*Marital status*				
Never married	42 (17.3)	68 (23.1)	33 (18.3)	143 (19.6)
Married/cohabiting	136 (56.0)	181 (61.5)	107 (59.5)	424 (58.3)
Separated/divorced	39 (16.0)	26 (8.9)	17 (9.4)	82 (12.9)
Widowed	26 (10.7)	19 (6.5)	23 (12.8)	68 (9.2)
*Ethnicity*				
Akan	56 (22.9)	86 (31.2)	3 (1.7)	145 (24.5)
Ga/Adangbe	86 (35.3)	85 (30.8)	156 (87.6)	327 (37.1)
Ewe	55 (22.5)	96 (34.8)	19 (10.7)	170 (26.3)
Other	47 (19.3)	9 (3.2)	0	56 (12.1)
*Educational status*				
No formal education	45 (18.0)	30 (10.2)	41 (22.8)	116 (15.3)
Basic education	147 (58.8)	165 (55.9)	107 (59.4)	419 (57.8)
Secondary/ SHS	37 (14.8)	65 (22.0)	21 (11.7)	123 (17.3)
Tertiary	21 (8.4)	35 (11.9)	11 (6.1)	67 (9.6)
*Religion*				
None	10 (4.1)	1 (0.4)	2 (1.1)	13 (2.5)
Islam	45 (18.2)	22 (7.5)	12 (6.7)	79 (13.4)
Christian	189 (76.5)	267 (91.1)	161 (90.0)	617 (82.9)
Other	3 (1.2)	3 (1.0)	4 (2.2)	10 (1.2)
*Average monthly Income*				
< GH₵ 200	53 (26.9)	35 (18.0)	45 (35.7)	133 (24.4)
GH₵ 200–GH₵ 499	74 (37.5)	68 (34.9)	47 (37.3)	189 (36.6)
GH₵ 500–GH₵ 999	47 (23.9)	57 (29.2)	20 (15.9)	124 (25.2)
GH₵ 1000 +	23 (11.7)	35 (17.9)	14 (11.1)	72 (13.8)
*Work status*				
Government employee	15 (7.1)	17 (6.4)	7 (4.6)	39 (6.6)
Non-government employee	160 (75.1)	190 (71.2)	110 (71.4)	460 (73.3)
Unemployed	26 (12.2)	42 (15.7)	23 (14.9)	91 (13.8)
Retired	12 (5.6)	18 (6.7)	14 (9.1)	44 (6.3)
*Health insurance status*				
Insured, Active	94 (38.9)	98 (33.2)	63 (35.4)	255 (36.4)
Insured, Expired	93 (38.4)	108 (36.6)	72 (40.4)	273 (37.9)
Not Insured	55 (22.7)	89 (30.2)	43 (24.2)	187 (25.7)

^*^ Weighted percentages

The Ga-Adangbe ethnic group formed majority (37.1%) of the respondents. On education, majority (57.8%) of the respondents only had basic education, with 15.4% not having any formal education.

A considerable number of the respondents (46.9%) had all their 32 teeth present. The metropolitan areas recorded a slightly higher amount of tooth loss compared to the other areas (Table 2).

**Table 2:** Distribution of oral conditions by socio-demographic characteristics

Characteristic	DMFT Mean (SE)	Prevalence n (%*)			
		Caries	Gingivitis	Periodontitis	Severe periodontitis
*Residence*					
Urban	1.83 (0.12)	195 (39.9)	443 (85.3)	235 (45.8)	71 (14.4)
Rural	1.81 (0.18)	91 (43.7)	175 (81.6)	103 (52.2)	22 (10.4)
*District type*					
Metropolitan	2.20 (0.18)	115 (45.8)	216 (86.1)	116 (46.2)	41 (16.3)
Municipal	1.53 (0.16)	96 (32.3)	245 (82.5)	142 (47.8)	32 (10.8)
Ordinary District	1.77 (0.19)	75 (41.4)	181 (86.7)	80 (44.2)	20 (11.1)
*Sex*					
Male	1.46 (0.13)	110 (36.6)	264 (86.3)	150 (48.9)	46 (16.2)
Female	2.08 (0.14)	176 (42.8)	354 (83.8)	188 (45.3)	47 (12.4)
*Age group*					
25–34	0.64 (0.09)	52 (23.7)	190 (82.2)	71 (29.8)	9 (4.9)
35–44	1.44 (0.16)	71 (40.8)	152 (88.1)	66 (40.4)	9 (6.0)
45–54	2.02 (0.23)	57 (41.6)	118 (84.0)	75 (49.1)	20 (12.7)
55–64	3.03 (0.34)	55 (51.8)	93 (85.8)	71 (68.1)	30 (30.4)
65–74	3.68 (0.48)	31 (60.5)	41 (80.5)	34 (69.6)	19 (44.7)
75 +	5.18 (0.84)	18 (89.6)	20 (92.3)	18 (75.1)	6 (15.5)
*Marital status*					
Never Married	0.76 (0.13)	40 (30.7)	123 (87.6)	53 (34.9)	5 (4.3)
Married/ Cohabiting	1.79 (0.13)	171 (41.5)	358 (83.9)	191 (45.3)	51 (12.6)
Separated / Divorced	2.29 (0.33)	31 (38.3)	67 (82.5)	41 (53.0)	14 (19.5)
Widowed	3.79 (0.45)	38 (55.2)	60 (87.6)	47 (72.2)	21 (34.1)
*Ethnicity*					
Akan	1.42 (0.21)	47 (34.3)	122 (84.7)	68 (45.0)	17 (10.8)
Ga/ Adangbe	1.89 (0.15)	129 (39.6)	279 (85.0)	151 (48.6)	44 (16.5)
Ewe	1.98 (0.22)	73 (43.1)	149 (89.3)	89 (53.5)	24 (15.2)
Other	2.09 (0.36)	27 (51.8)	42 (74.0)	20 (34.3)	7 (12.6)
*Educational status*					
No formal education	2.65 (0.31)	54 (49.7)	97 (82.8)	62 (57.7)	19 (17.8)
Basic education	1.91 (0.14)	172 (41.5)	357 (85.4)	201 (48.9)	60 (16.5)
Secondary/ SHS	1.11 (0.17)	36 (31.1)	101 (81.0)	44 (34.3)	7 (4.7)
Tertiary	1.18 (0.27)	22 (36.5)	60 (91.6)	28 (37.7)	6 (8.1)
*Religion*					
None	2.77 (0.78)	7 (64.2)	12 (90.8)	10 (72.5)	2 (18.4)
Islam	1.98 (0.31)	30 (40.4)	62 (76.4)	28 (33.5)	6 (8.0)
Christian	1.73 (0.11)	237 (39.1)	527 (85.9)	290 (48.0)	83 (14.7)
Other	2.4 (0.70)	6 (57.0)	9 (89.3)	7 (75.4)	1 (18.6)
*Average monthly Income*					
< GH₵ 200	2.16 (0.26)	53 (43.4)	117 (90.6)	64 (52.4)	17 (13.3)
GH₵ 200–GH₵ 499	1.74 (0.18)	74 (38.6)	154 (81.1)	76 (43.4)	21 (13.6)
GH₵ 500–GH₵ 999	1.61 (0.24)	49 (42.2)	102 (83.2)	56 (43.6)	19 (17.8)
GH₵ 1000 +	1.68 (0.33)	25 (35.6)	63 (83.9)	27 (33.4)	9 (10.5)
*Work status*					
Government	0.95 (0.29)	8 (26.0)	31 (82.2)	18 (45.1)	4 (12.2)
Non-government	1.79 (0.13)	187 (42.2)	395 (85.6)	212 (47.0)	56 (13.9)
Unemployed	0.89 (0.17)	20 (21.5)	74 (81.0)	39 (41.1)	8 (10.1)
Retired	4.05 (0.54)	32 (78.6)	37 (83.6)	30 (70.6)	9 (19.5)
*Health insurance status*					
Insured, Active	2.40 (0.20)	108 (43.4)	216 (86.7)	130 (50.5)	36 (14.3)
Insured, Expired	1.67 (0.15)	114 (42.8)	231 (82.9)	106 (39.5)	27 (10.6)
Not Insured	1.25 (0.15)	59 (33.5)	160 (85.8)	96 (53.1)	28 (18.4)

^*^ Weighted percentages

The prevalence of untreated caries was 40.4%, while 26.7% had retained roots. These showed great variation among the different districts, with the metropolitan districts showing a higher prevalence and the municipal districts having the least (Table 3).

**Table 3:** Distribution of Oral Health variables by district type and residence

Characteristic	Metropolitan n (%*) 251 (55.7)	Municipal n (%*) 297 (37.9)	Ordinary District n (%*) 181 (6.40)	Urban n (%*) 519 (86.3)	Rural n (%*) 210 (13.7)	Pooled n (%*) 729 (100)
*Disease prevalence*						
Gingivitis	216 (86.1)	245 (82.5)	181 (86.7)	443 (85.3)	175 (81.6)	618 (84.8)
Periodontitis	116 (46.2)	142 (47.8)	80 (44.2)	235 (45.8)	103 (52.2)	338 (46.7)
Severe Periodontitis	41 (16.3)	32 (10.8)	20 (11.1)	71 (14.4)	22 (10.4)	93 (13.9)
Caries	115 (45.8)	96 (32.3)	75 (41.4)	195 (39.9)	91 (43.7)	286 (40.4)
Retained roots	76 (30.3)	63 (21.2)	50 (27.6)	127 (26.3)	62 (28.8)	189 (26.7)
Mean teeth present	29.5 (0.2)	30.4 (0.2)	30.2 (0.2)	30.0 (0.1)	30.2 (0.2)	30.02(0.2)
OH coverage	3 (3.2)	7 (6.9)	4 (5.4)	9 (4.7)	5 (4.9)	14 (4.7)
*Oral health characteristics*						
Dentist visit						
Ever	99 (39.8)	84 (28.5)	39 (21.5)	170 (35.6)	52 (26.2)	222 (34.3)
Never	150 (60.2)	211 (71.5)	142 (78.5)	345 (64.4)	158 (73.8)	503 (65.7)
Last dental visit						
Within last 6 months	6 (6.7)	7 (8.8)	2 (5.6)	13 (8.0)	2 (2.0)	15 (7.3)
Within last one year	7 (7.8)	10 (12.5)	4 (11.1)	17 (9.8)	4 (4.0)	21 (9.5)
1- 5 years ago	17 (18.9)	9 (11.3)	5 (13.9)	24 (16.4)	7 (15.0)	31 (16.2)
> 5 years ago	60 (66.7)	54 (67.5)	25 (69.4)	103 (65.9)	36 (76.2)	139 (76.1)
Frequency of dental visit						
Once a year	7 (7.3)	8 (10.1)	2 (5.3)	13 (7.9)	4 (9.5)	17 (8.1)
2 X a year or more	5 (5.2)	3 (3.8)	1 (2.6)	9 (5.2)	0	9 (4.7)
When problem	84 (87.5)	68 (86.1)	35 (92.1)	141 (86.9)	46 (90.5)	187 (87.3)
Ever had an oral practitioner clean your teeth?						
Yes	17 (7.1)	16 (5.5)	6 (3.4)	29 (6.4)	10 (5.4)	39 (6.3)
No	221 (92.9)	276 (94.5)	170 (96.6)	474 (93.6)	193 (94.6)	667 (93.7)
Tooth cleaning times per day						
Once	76 (31.9)	83 (29.1)	43 (25.9)	154 (31.8)	48 (22.3)	202 (30.5)
Twice or more	161 (67.6)	201 (70.5)	122 (73.5)	338 (67.9)	146 (76.5)	484 (69.1)
When I remember	1 (0.4)	1 (0.4)	1 (0.6)	1 (0.3)	2 (1.3)	3 (0.4)
Main method of tooth cleaning						
Toothbrush	172 (72.6)	263 (91.0)	151 (84.8)	411 (79.5)	174 (86.3)	586 (80.5)
Chewing sponge	50 (21.1)	18 (6.2)	12 (6.7)	67 (15.8)	13 (6.2)	80 (5.1)
Chewing stick	15 (6.3)	8 (2.8)	15 (8.4)	20 (4.7)	18 (7.5)	38 (14.4)
Texture of toothbrush						
Very hard (smokers)	21 (9.2)	16 (5.5)	14 (8.2)	37 (7.9)	14 (6.6)	51 (7.7)
Hard	57 (25.0)	91 (31.5)	77 (45.0)	145 (27.8)	80 (35.6)	225 (28.9)
Medium	98 (43.0)	117 (40.5)	50 (29.2)	198 (41.5)	67 (38.4)	265 (41.1)
Soft	52 (22.8)	65 (22.5)	30 (17.5)	109 (22.8)	38 (19.4)	147 (22.3)
Health Insurance Status						
Insured, Active	94 (38.8)	98 (33.2)	63 (35.4)	190 (37.9)	65 (27.4)	255 (36.5)
Insured, Expired	93 (38.4)	108 (36.6)	72 (40.5)	187 (36.7)	86 (45.2)	273 (37.9)
Not Insured	55 (22.7)	89 (30.2)	43 (24.2)	132 (25.4)	55 (27.5)	187 (25.7)
Alcohol use						
Never	118 (53.6)	129 (46.2)	82 (51.6)	233 (51.4)	96 (46.0)	329 (50.6)
Formerly	18 (8.2)	35 (12.5)	8 (5.0)	43 (9.1)	18 (13.4)	61 (9.7)
Drink Regularly	24 (10.9)	10 (3.6)	6 (3.8)	34 (8.3)	6 (3.1)	40 (7.6)
Drink Occasionally	60 (27.3)	105 (37.6)	63 (39.6)	155 (31.2)	73 (37.6)	228 (32.1)
Tobacco use						
Non smoker	229 (95.0)	274 (95.8)	166 (93.3)	474 (95.3)	195 (94.8)	669 (95.2)
Former smoker	6 (2.5)	7 (2.5)	9 (5.1)	16 (2.6)	6 (3.0)	22 (2.6)
Current smoker	6 (2.5)	5 (1.8)	3 (1.7)	11 (2.2)	3 (2.2)	14 (2.2)

^*^Weighted percentages

Gingivitis was very prevalent, being higher in the ordinary districts and least in the municipal districts. Periodontitis, though higher in the municipal areas was more severe in the metropolitan areas (Table 3).

Only 34.3% reported ever visiting a dentist with the proportions being higher in the metropolitan areas. Also, of the 269 respondents who reported problems with their mouth, only 4.7% had utilized an oral health facility. With oral hygiene practises, the metropolitan areas reported less frequency in oral hygiene practices in a day and also a lower proportion of toothbrush use (Table 3).

The prevalence of oral conditions varied greatly among the different EA’s with Avenor having a very high prevalence of oral diseases (Table 4).

**Table 4:** Distribution of oral characteristics by enumeration area

Enumeration area	Mean teeth present Mean (SE)	Ever visited a dentist n (%)	DMFT Mean (SE)	Prevalence %(SE)					Oral problems over past 12 months n (%)	OH Utilization % (SE)
				Caries	Retained roots	Gingivitis	Periodontitis	Severe Periodontitis		
Achimota^δ^	29.2 (0.57)	13 (37.1)	2.2 (0.46)	54.3 (0.08)	34.3 (0.08)	94.3 (0.04)	68.6 (0.08)	17.1 (0.06)	14 (40.0)	0
Adenta^€^	30.6 (0.40)	19 (47.5)	1.5 (0.42)	34.2 (0.07)	14.6 (0.06)	85.4 (0.06)	53.7 (0.08)	12.2 (0.05)	16 (39.0)	12.5 (0.08)
Ashiaman^€^	30.4 (0.42)	12 (30.0)	2.0 (0.47)	45.0 (0.08)	30.0 (0.07)	87.5 (0.05)	50.0 (0.08)	12.5 (0.05)	11 (27.5)	9.1 (0.09)
Akporman^€^	30.6 (0.38)	8 (23.5)	1.4 (0.33)	41.2 (0.08)	11.8 (0.06)	73.5 (0.08)	58.8 (0.08)	8.8 (0.05)	14 (42.4)	0
Accra New Town^δ^	29.7 (0.46)	14 (38.9)	2.3 (0.43)	47.2 (0.08)	27.8 (0.08)	72.2 (0.08)	25.0 (0.07)	5.6 (0.04)	15 (41.7)	0
Avenor^δ^	27.8 (0.71)	17 (46.0)	3.6 (0.64)	59.5 (0.08)	40.5 (0.08)	86.5 (0.06)	48.7 (0.08)	18.9 (0.05)	16 (43.2)	6.3 (0.06)
Ayikuma^Ω^	29.0 (0.54)	15 (44.1)	2.9 (0.50)	61.8 (0.08)	41.2 (0.08)	73.5 (0.08)	38.2 (0.08)	17.7 (0.07)	29 (85.3)	6.9 (0.05)
Dawa^Ω^	31.3 (0.26)	3 (8.6)	0.6 (0.22)	14.3 (0.06)	14.3 (0.06)	91.4 (0.05)	40.0 (0.08)	5.7 (0.04)	11 (32.4)	0
Dome^€^	29.7 (0.62)	9 (26.5)	2.0 (0.62)	32.4 (0.08)	26.5 (0.08)	91.2 (0.05)	32.4 (0.08)	11.8 (0.06)	11 (33.3)	9.1 (0.09)
Gbawe^€^	30.0 (0.52)	8 (23.0)	1.9 (0.45)	31.7 (0.08)	20.2 (0.07)	71.2 (0.08)	33.8 (0.08)	10.8 (0.06)	9 (28.0)	0
Katapor^€^	29.8 (0.55)	11 (32.4)	2.2 (0.52)	47.1 (0.09)	44.1 (0.09)	85.3 (0.06)	52.9 (0.09)	11.8 (0.06)	14 (41.2)	7.1 (0.07)
Korle Gonno^δ^	29.8 (0.58)	16 (39.0)	1.5 (0.37)	36.6 (0.08)	26.8 (0.07)	87.8 (0.05)	43.9 (0.08)	17.1 (0.06)	15 (37.5)	0
Lolonya^Ω^	30.0 (0.57)	9 (23.7)	2.1 (0.47)	52.6 (0.08)	42.1 (0.08)	89.5 (0.05)	52.6 (0.08)	13.2 (0.06)	15 (41.7)	13.1 (0.09)
New Achimota^€^	30.6 (0.41)	10 (23.8)	1.0 (0.34)	14.0 (0.05)	11.6 (0.05)	95.4 (0.03)	74.4 (0.07)	14.0 (0.05)	14 (32.6)	7.1 (0.07)
New Aplaku^€^	31.1 (0.34)	7 (18.4)	0.5 (0.20)	15.8 (0.06)	13.2 (0.06)	68.4 (0.08)	23.7 (0.07)	7.9 (0.04)	13 (34.2)	7.7 (0.07)
Old Ningo^Ω^	30.4 (0.45)	6 (15.4)	1.5 (0.38)	35.9 (0.08)	18.0 (0.06)	92.3 (0.04)	38.5 (0.08)	12.8 (0.05)	18 (47.4)	0
Osu^δ^	30.3 (0.44)	17 (53.1)	1.8 (0.42)	45.5 (0.09)	15.2 (0.06)	93.9 (0.04)	33.3 (0.08)	12.1 (0.06)	14 (42.4)	0
Russia^δ^	30.0 (0.47)	12 (35.3)	1.9 (0.48)	44.1 (0.09)	32.4 (0.08)	82.4 (0.07)	41.2 (0.08)	11.8 (0.06)	11 (32.4)	18.2 (0.12)
Tamatokou^Ω^	30.2 (0.44)	6 (17.1)	1.8 (0.43)	42.9 (0.08)	22.9 (0.07)	85.7 (0.06)	51.4 (0.09)	5.7 (0.04)	1 (2.9)	0
Tema New Town^δ^	29.7 (0.51)	10 (30.3)	2.1 (0.52)	35.3 (0.08)	35.3 (0.08)	85.3 (0.06)	61.8 (0.08)	29.4 (0.08)	8 (24.2)	0

δ Metropolitan € Municipal Ω District

Though disease prevalence differed in the different enumeration areas, a multivariate analysis showed no significant difference in caries and periodontal disease prevalence in the different district types and by residence (Table 5).

**Table 5:** Variation in oral disease by district type, residence and enumeration area

Characteristic	Caries		Periodontitis	
	Crude OR (95%CI)	†Adjusted OR (95%CI)	Crude OR (95%CI)	†Adjusted OR (95%CI)
*District type*				
Metropolitan	1.00	1.00	1.00	1.00
Municipal	0.59 (0.40–0.86)	0.68 (0.45–1.01)	1.07 (0.76–1.49)	1.32 (0.91–1.90)
District	0.75 (0.49–1.15)	0.81 (0.52–1.26)	0.92 (0.63–1.35)	1.01 (0.66–1.51)
*Residence*				
Urban	1.00	1.00	1.00	1.00
Rural	0.99 (0.66–1.51)	1.05 (0.69–1.60)	1.29 (0.89–1.87)	1.40 (0.95–2.08)
*Enumeration Area*				
Achimota	1.00	1.00	1.00	1.00
Adenta	0.55 (0.21–1.45)	0.61 (0.22–1.66)	0.53 (0.21–1.36)	0.50 (0.18–1.37)
Ashiaman	0.64 (0.25–1.68)	0.71 (0.26–1.92)	0.46 (0.18–1.18)	0.40 (0.15–1.10)
Akporman	0.72 (0.27–1.93)	0.76 (0.27–2.10)	0.65 (0.24–1.76)	0.64 (0.23–1.75)
Accra New Town	0.85 (0.32–2.22)	0.83 (0.30–2.31)	0.15 (0.05–0.43)	0.10 (0.03–0.31)
Avenor	1.28 (0.50–3.26)	1.10 (0.41–2.93)	0.43 (0.17–1.14)	0.24 (0.09–0.66)
Ayikuma	1.05 (0.40–2.75)	1.23 (0.44–3.43)	0.28 (0.10–0.77)	0.24 (0.09–0.68)
Dawa	0.14 (0.04–0.55)	0.17 (0.42–0.67)	0.31 (0.11–0.82)	0.36 (0.13–0.97)
Dome	0.46 (0.16–1.31)	0.50 (0.17–1.45)	0.22 (0.08–0.60)	0.17 (0.05–0.51)
Gbawe	0.60 (0.22–1.66)	0.61 (0.21–1.68)	0.23 (0.08–0.65)	0.17 (0.06–0.51)
Katapor	0.54 (0.19–1.50)	0.58 (0.20–1.68)	0.52 (0.19–1.38)	0.45 (0.16–1.29)
Korle Gonno	0.62 (0.24–1.61)	0.65 (0.24–1.76)	0.36 (0.14–0.92)	0.26 (0.10–0.69)
Lolonya	0.69 (0.26–1.82)	0.63 (0.22–1.76)	0.51 (0.20–1.33)	0.37 (0.13–1.06)
New Achimota	0.15 (0.04–0.53)	0.19 (0.05–0.67)	1.33 (0.49–3.59)	1.64 (0.59–4.58)
New Aplaku	0.08 (0.02–0.40)	0.11 (0.02–0.54)	0.14 (0.50–0.40)	0.19 (0.07–0.54)
Old Ningo	0.52 (0.19–1.39)	0.50 (0.18–1.43)	0.29 (0.11–0.75)	0.18 (0.07–0.50)
Osu	0.86 (0.32–2.29)	0.92 (0.33–2.57)	0.23 (0.08–0.63)	0.22 (0.08–0.63)
Russia	0.54 (0.19–1.50)	0.57 (0.20–1.58)	0.32 (0.12–0.86)	0.26 (0.09–0.72)
Tamatokou	0.60 (0.22–1.63)	0.65 (0.23–1.83)	0.49 (0.18–1.29)	0.46 (0.16–1.29)
Tema New Town	0.32 (0.11–0.98)	0.31 (0.09–1.00)	0.74 (0.27–2.00)	0.49 (0.17–1 42)

^†^ Multivariate analysis was carried out to account for age and sex

## Discussion

This study describes the participant characteristics, study design and methods used in carrying out an oral health survey among adults in the GAR. It is the first comprehensive study covering the region to assess the prevalence, and correlates of oral disease using NHANES and CDC/AAP guidelines. Its strength is in the large sample size, with a population-based random sample selection and the comprehensive clinical examination used. A number of methodological procedures along with findings from the different districts and localities are presented.

The study was a cross-sectional study, measuring associations at one point in time, and was therefore unable to establish causal relationships.

Dental diseases are prevalent in the GAR with variation within the different districts and localities. The municipal districts had lower prevalence’s of most disease conditions, they also had more teeth present and better oral health coverage. Ironically, the metropolitan areas which have the highest number of dental clinics and dentists and better insurance coverage had more dental disease and lower oral health coverage. Sisson [21] proposes several reasons for these inequalities however, one can conclude from these findings that availability of facilities and the means to assess them alone is not enough to significantly result in better oral health outcomes. A previous study on edentulism in Ghana [22] also reported no association with oral health coverage and health insurance. Though the causes of oral diseases are largely genetic and microbiological, behavioural and social factors such as community cultural values, health beliefs, attitudes, and knowledge of the dental care delivery system also greatly influence oral health [23].

The variation in disease prevalence was more pronounce for dental caries and its consequences than for periodontal disease. Though many factors may contribute to the differences in oral diseases across the different geographical locations, the availability of natural fluoride in the drinking water may play a vital role. This is because apart from sugar consumption, both caries and periodontal disease share similar risk factors. Thus, the naturally occurring fluorides which protect the teeth from the acid attack may account for this. Antwi et al. [24], reported a generally low fluoride content of the freshwater system in Ghana with none of eight rivers surveyed having fluoride levels greater than 0.3 ppm. The boreholes were however richer in fluoride. This may also account for the wide variation in disease prevalence in some of the EA’s. Further research may need to be undertaken to relate this to each community’s oral health needs so effective interventions can be developed.

This study revealed that about 66.0% of the population had never visited a dentist in their lifetime, a high figure when compared with 22.0% in Benin city, Nigeria [25] but similar to 71.0% in Rwanda [26]. An earlier study among adolescents in one of the districts in GAR [27] however reported 84.0% had never been to a dentist. Thus, generally oral health utilization in this region is very low. The unmet need for oral health was also high (95.0%). A study [7] assessing unmet need for oral health services among the elderly in China, Ghana, and India, reported a prevalence of 80.0% for Ghana compared with 60.0% and 62.0% for China and India respectively. A study in Burkina Faso [23] reported 72.0%. Ghana and especially GAR has very well-qualified dentists. However, one must note that many African communities have their own traditional oral health practices including traditional healers and self-medication which may account for this low utilization [28]. This notwithstanding, majority of the population still had all of their teeth still present.

With regards to oral hygiene practices, while majority (70.0%) reported cleaning their teeth twice or more a day, quite a huge number were still using mainly traditional tooth cleaning methods e.g., the chewing stick (miswak). This has been a common, culturally accepted practice in Ghana [29] with some still preferring them over the toothbrush. These traditional methods apart from their mechanical cleaning action, contain antibacterial agents that are helpful with oral hygiene. The WHO therefore recommends that communities can be encouraged to use them in combination with fluoride toothpastes [28].

On disease prevalence, though caries prevalence may be considered low, the high levels of untreated caries including retained roots is a great cause for concern. The prevalence of periodontitis was comparable to that worldwide, it was however more severe. This raises priority areas for policy makers to consider to improve the oral health of Ghanaians. Oral health has generally not been prioritized in Ghana however it is essential to the achievement of Universal Health Coverage especially due to the high and increasing prevalence of oral diseases and their impact on general health. Unfortunately, the proportion of people obtaining oral health care is alarmingly low despite efforts at improving access including increasing number of trained dentists and clinics, and revamping the national health insurance. This study and others [22] in Ghana, has shown that availability of dentists and access to dental services alone does not appear to be the most important determinant of better oral health outcomes. Effective action to tackle these inequalities can only be developed when the underlying causes are identified and understood. We propose that though treatment services are important in maintaining oral health, an emphasis be placed on prevention to achieve sustainable oral health improvements, and to reduce oral health inequalities especially since only a small proportion of the population regularly utilize oral health services. Implementing appropriate and targeted education programs directed at risk populations may also be needed. Furthermore, in most African countries, strong environmental, socio-political, cultural and behavioural forces determine people’s beliefs and conceptions of health and disease [30]. Sisson [21] argues that epidemiological methods identify and quantify risk factors for disease, providing a basis for describing but not explaining disease. It thus disconnects individuals from their social context, neglecting broad social factors such as how individuals live their lives and what influences their lifestyle decisions. The impact of social interaction on oral health of this population should therefore be explored and harnessed to improve outcomes.

## Conclusion

Regional inequalities in oral health exist in the GAR. The WHO Regional Committee for Africa proposed a strategy to improve oral health. Two of its guiding principles included focusing interventions on the district and its communities and enlisting the participation of communities in oral health activities. This study assessed the oral health of residents in the GAR. It identified the magnitude and distribution of oral diseases and assessed behaviour and practices that may be promoting them while engaging these communities. It therefore provides district and community level data to achieve that.

## Data Availability

The datasets used and/or analysed during the current study are available from the corresponding author on reasonable request.

## Abbreviations

AAP: American Academy of Periodontology
BMI: Body mass index
BP: Blood pressure
BOP: Bleeding on probing
CDC: Centers for Disease Control and Prevention
CEJ: Cemento-enamel junction
CAL: Clinical attachment loss
EA: Enumeration area
FGM: Free gingival margin
NHANES: National Health and Nutrition Examination Survey
PPD: Probing pocket depth
UGDS: University of Ghana Dental School
WHO: World Health Organization
WHR: Waist hip ratio
